# Nutritional predictors of lymphatic filariasis progression: Insights from a machine learning approach

**DOI:** 10.1371/journal.pone.0320640

**Published:** 2025-03-26

**Authors:** Emmanuel Kobla Atsu Amewu, Benedicta Amaglo, Priscilla Osei-Poku, Arnold Abakah, Abdul Latif Koney Shardow, Lauri Wright, Alexander Kwarteng

**Affiliations:** 1 Kumasi Centre for Collaborative Research in Tropical Medicine, KCCR, Kwame Nkrumah University of Science and Technology, Kumasi, Ghana; 2 Department of Biochemistry and Biotechnology, Kwame Nkrumah University of Science and Technology, Kumasi, Ghana; 3 College of Public Health, University of South Florida, Tampa, United States of America; Instituto Leonidas e Maria Deane / Fundacao Oswaldo Cruz, BRAZIL

## Abstract

Lymphatic filariasis (LF) is a mosquito-borne neglected tropical disease that causes disfiguring of the affected extremities, often leading to permanent disability and stigma. Described as a disease of poverty, the impact of socioeconomic indicators such as nutrition on LF remains largely unexplored. This cross-sectional study investigates nutritional predictors implicated in the progression of LF using machine learning methods in the Ahanta West Municipality, Ghana. There were 109 participants with a mean age of 50.72±13.8, and three-quarters being females. Only 14 (12.8%) each had comorbidities or LF-related wounds. Nutrition risk assessment showed 70.7% of participants were either malnourished or at risk of being malnourished. The prevalence of anemia was 84.0%. Dietary assessment indicates marked macro- and micronutrient intake with 98.2% protein, 75.2% fat, and 73.4% carbohydrate inadequacies. There were inadequate intakes of minerals: Calcium (100%), Potassium (91.7%) and Zinc (91.7%); and vitamins: Vitamin B12 (81.7%), Vitamin C (75.2%), Niacin (70.6%), and Vitamin B6 (68.8%). The decision tree and random forest models show vitamins C and K and blood pressure as the most important predictors of LF progression. Other predictors include body mass index, anemia, folate, and age. These findings suggest that maintaining healthy blood pressure and adequate intake of vitamins C and K may slow LF progression. This highlights the importance of nutritional intervention and underscores the need for integrated approaches that address nutritional deficiencies and LF management strategies.

## Introduction

Lymphatic filariasis (LF) is one of the neglected tropical diseases (NTDs), which cause chronic, debilitating, and disfiguring pathologies. The disease, caused by *Wuchereria bancrofti* or *Brugia* spp., is transmitted through mosquito bites [1]. The disease affects more than 50 million people, with more than 36 million living with chronic manifestations. This makes LF a significant public health issue with mental health and socioeconomic burdens for sufferers [2]. Over 1.1 billion people are at risk of LF in the tropical and subtropical regions of Asia, Africa, the western Pacific, and portions of South America and the Caribbean [3]. Individuals living in endemic areas are typically infected during childhood; the disease can persist for decades [4]. Epidemiological studies have identified several risk factors associated with the progression of LF, including age, gender, and socioeconomic status [5–8]. Given the established link between infection and nutritional status, exploring how specific nutritional deficiencies affect LF progression is crucial.

The impact of nutritional status on the progression of LF is an important area of research, yet it remains largely unexplored. Evidence suggests that addressing nutritional deficiencies and promoting healthy eating habits may be necessary for controlling the progression of the disease and improving the outcomes for the affected individuals [9]. This suggests that the interplay between nutrition and the host’s immune response may play a significant role in LF progression.

Chronic infections, such as LF, can profoundly impact an individual’s nutritional status and overall metabolic health [10]. Chronic undernutrition and infection are closely interlinked, with the combination of the two further weakening immune response. Primary malnutrition, caused by inadequate macronutrients or selected micronutrients, can also lead to clinically significant immune deficiency and increased susceptibility to infections in children [11]. Micronutrient deficiencies, such as those in vitamins A, D, E, and K, and minerals like zinc, iron, and selenium, can also impair immune function [12]. The additional role of vitamin K in cardiovascular health is also well established. Vitamin C, with deficiency associated with poor socioeconomic background, plays critical role in the repair of damaged tissues such as the skin.

There is limited research exploring the impact of nutritional deficiencies on the progression of LF. The additional subjective and non-consensus nature of nutrition risk assessment makes identifying at-risk patients for appropriate intervention challenging. Existing literature suggests that certain micronutrients, such as vitamin A, zinc, and iron, may play a role in the body’s immune response to the parasitic infection either by exacerbating it or reducing the risk of infection [12,13]. In the context of LF, the accumulation of lymphatic fluids is likely to impact fluid balance, and vitamins such as C and K are likely to impact repair of damaged skin and cardiovascular health respectively. Therefore, there is a need to understand the specific mechanisms by which micronutrients influence the progression of LF. This study uses machine learning models to identify key nutritional and physiological predictors of LF from early to late/advanced stage.

## Materials and methods

### Study design

A cross-sectional study was conducted among individuals clinically diagnosed with LF in the Ahanta West District, southeastern Ghana. Participants were recruited based on LF pathology and categorized into early stage (1-3) and advanced stage (4-7) based on the seven-stage Dreyer classification system [14]: 1) swelling is reversible overnight; 2) swelling is not reversible overnight; 3) presence of shallow skin folds; 4) presence of knobs/bumps/lumps; 5) presence of deep skin folds; 6) presence of ‘mossy foot’ and bad odor; 7) unable to care for self and perform daily activities. Participants in this study, carried out between February 2022 and June 2022, were from stages 1 to 6.

### Study site and population

The study was conducted in seven LF-endemic communities in the Ahanta West District, viz Akatakyi, Ampatano, Asemkow, Busia, Butre, Dixcove, and Princess Town. Ahanta West is one of the 22 districts in the Western region, with a total area of about 636 km^2^ and a population of 106,000. The region experiences a relatively high humidity level, which averages between 75-85% during the rainy season and 70-80% during the dry season. This humid weather provides a conducive environment for the proliferation of mosquitoes, the transmitting vector of LF.

### Sample size and sampling technique

Sample size, computed with G * Power 3.1 for linear multiple regression with effect size of 0.15, alpha of 0.05, power of 0.80, and with 25 predictors, was 172. In total, 109 study participants (LF patients) were conveniently sampled based on availability in the communities visited. This approach was adopted based on logistical and prevailing situation on the field to ensure most participants in the communities were included. Consequently, power analysis based on the actual sample size gave a statistical power of 0.62. Generalization of findings from this study should be done cautiously.

### Approval and ethics considerations

This study was approved by the Committee on Human Research Publication and Ethics at the School of Medical Sciences and Dentistry, KNUST, Kumasi, with approval number CHRPE/AP/062/22, and the Ahanta West Municipal Health Directorate. Participants were recruited between 16th February 2022 and 15^th^ February 2023. In addition, written consent was obtained from all participants after they had been adequately informed about the study prior to recruitment. All personally identifiable information were removed, and unique participant codes were assigned. The study was conducted in accordance with Helsinki’s declaration.

### Inclusivity in global research

Additional information regarding the ethical, cultural, and scientific considerations specific to inclusivity in global research is included in the Supporting Information (S2 Checklist)

### Data collection

#### Sociodemographic and medical history.

To understand the socioeconomic context and its potential influence on the nutritional and health status of participants, a structured questionnaire was used to collect data on sociodemographic characteristics, including age, gender, marital status, education level, and occupation. Participants were asked about their medical history, including any previous diagnoses, ongoing health conditions, and the presence of LF-related wounds.

#### Dietary history.

A 3-day, 24-hour dietary recall from the Center for Disease Control (CDC)’s dietary interview component and the Food and Agriculture Organization (FAO) [15] was used to assess dietary history. Participants were asked to recall all foods and beverages consumed over the past 24 hours on three days, including one weekend. The recall process involved visual aids and handy measures to estimate portion sizes. Completed diet histories were analyzed using the Nutrient Analysis Template [16] to estimate energy, macronutrients, and micronutrient intakes. The average daily intake was compared to the Recommended Dietary Allowances (RDA) and Acceptable Macronutrient Distribution Ranges (AMDR).

#### Anthropometric measurements.

Height was measured to the nearest 0.1 cm using the Seca® stadiometer, weight to the nearest 0.1 kg, and body mass index (BMI) computed using the OMRON^®^ body composition analyzer. Body and visceral fats were also measured with the OMRON^®^ body composition analyzer.

#### Waist and hip circumferences.

To assess central obesity and to determine participants’ risk of metabolic complications related to their nutritional status, waist and hip circumferences were measured using a non-stretchy tape measure. The waist-to-hip ratio (WHR) was calculated by dividing the waist circumference by the hip circumference.

#### Blood pressure.

Blood pressure measurements were taken using an OMRON® digital blood pressure monitor. Three readings were taken at rest, and the average systolic and diastolic blood pressure values were recorded for each participant.

#### Physical activity.

The physical activity level of each participant was assessed using the International Physical Activity Questionnaire (IPAQ). Frequency, activity (walking, moderate and vigorous intensity) and duration were assessed based on the number of days per week each participant spent doing that activity.

#### Hematological assessment.

Hemoglobin levels were measured using the Hemocue 2.1 system. Blood samples were collected by pricking the finger with a lancet and allowing a small amount of blood to be drawn into a disposable microcuvette and then placed into the Hemocue 2.1 to analyze the sample and display the hemoglobin level on its screen. This was used to determine the prevalence of anemia among participants. Anemia was defined according to WHO criteria based on the hemoglobin levels adjusted for sex.

### Outcome variable

The primary outcome variable was the clinical stage of LF, categorized as early (stages 1-3) and advanced (stages 4-6) based on the Dreyer LF staging.

### Subjective Global Assessment (SGA) for nutrition status assessment

This study used the Mini Nutritional Assessment Short-Form (MNA-SF) to screen participants for nutritional risk [17]. The MNA-SF is a validated tool consisting of six items that assess key aspects of nutrition. Adjustments were made to adapt the MNA-SF scoring system based on the specific data available for this research.

The first criterion of the MNA-SF evaluates changes in food intake and is scored as 0 for a severe decrease, 1 for a moderate decrease, and 2 for no decrease in food intake. For this study, a severe decrease in intake was defined as consuming less than 75% of estimated energy needs, a moderate decrease as 76-90% of estimated needs, and no decrease as more than 90% of estimated needs. The second MNA-SF criterion assesses weight loss, with scores ranging from 0 for weight loss greater than 3 kg, 1 for unknown weight loss, 2 for weight loss between 1-3 kg, and 2 for no weight loss. In this study, weight loss was evaluated by changes in body fat percentage Branco *et al*. (2018) proposed a normative table for body fat percentages (see Table 1) [18]. Weight loss of more than 3 kg was categorized as a “poor” body fat percentage, weight loss between 1-3 kg as “below average,” and no weight loss as “average” or above. Mobility, the third MNA-SF criterion, is typically scored based on the participant’s ability to move. It assigns a score of 0 for those bed or chair-bound, 1 for those able to get out of bed/chair but who do not go out, and 2 for those who are able to go out. For this research, mobility was assessed using an activity-based classification: low activity scored 0, moderate activity scored 1, and high activity scored 2. The fourth criterion assesses psychological stress or the presence of an acute disease within the past three months, with a score of 0 for participants experiencing stress or illness and 2 for those who did not. In this study, acute disease was defined as being in LF stage 4 or higher or the presence of an active wound. The fifth criterion of the MNA-SF, neuropsychological problems could not be determined due to a lack of relevant data. Therefore, this item was excluded from the scoring for this study. The final criterion assesses body mass index (BMI) and is scored as 0 for a BMI of less than 19, 1 for a BMI between 19 and 20, 2 for a BMI between 21 and 22, and 3 for a BMI of 23 or higher.

**Table 1:** Normative body fat percentages [18].

Category	Male	Female
Low	8.9–12.5	18.7–23.2
Below Normal	12.6–17.6	23.2–28.7
Normal	17.6–25.3	28.8–35.7
Above Normal	25.4–35.1	35.8–42.9
Excessive	35.2–43	43–49.1
Very Excessive	43.1–49.4	49.2–52.1
Extremely Excessive	49.5 >	52.2 >

The total score for the MNA-SF typically ranges from 0 to 14, with higher scores indicating better nutritional status. Due to the absence of data for the neuropsychological problem criterion, the maximum score was adjusted to 12. Total scores were then categorized as follows: 0-5 indicating malnourishment, 6-9 indicating risk of malnutrition, and 10-12 indicating normal nutritional status. These adjustments allowed for the tool’s use in evaluating nutritional risk based on the available data.

### Machine learning approach

Machine learning techniques, decision trees, and random forests were employed in this study to explore complex, non-linear relationships between multiple nutritional and physiological variables, which may be lacking in traditional statistical methods. These models effectively handle non-linear relationships, accommodating complex interactions between variables, and providing interpretable outputs. The random forest algorithm builds multiple decision trees to classify individuals and averages their results. Categorical variables (factors) with single levels were identified and removed before model fitting. Additionally, we ensured consistency between the training and test datasets by aligning factor levels. Any categorical variable in the test data with levels absent in the training data was either combined with similar levels or assigned to an “Other” category. For categorical variables with rare levels (i.e., levels that appeared in fewer than a defined threshold of observations), we combined these levels into a new category labeled “Other.” The threshold for determining rare levels was set at five observations based on the distribution of levels in the dataset. The dataset was randomly partitioned into a training set (80%) and a test set (20%) using stratified sampling based on the outcome variable, LF stage, to ensure that both the training and test sets had similar distributions of categorical variables and outcome classes. Stratified sampling was performed to ensure that each class of the outcome variable was proportionally represented in both sets. After data preprocessing, we trained a random forest model using the prepared training dataset. Predictions were made on the test dataset, ensuring that factor levels in the test data were aligned with those in the training set to prevent any issues with contrasts or missing levels. To prevent overfitting and improve performance, we applied pruning techniques to the decision tree, reducing model complexity while maintaining predictive accuracy. Hyperparameter tuning for the decision tree involved optimizing parameters such as maximum depth and minimum split size. For the random forest model, we performed cross-validation with grid search to select optimal hyperparameters, including the number of trees (ntree) and the number of variables at each split (mtry). Model performance was evaluated using accuracy, precision, recall, F1-score, and AUC-ROC, ensuring robustness and generalizability.

### Data analysis

Categorical variables were summarized as frequencies and percentages, and continuous variables were summarized using means and standard deviations. Chi-square and Fisher’s exact tests were used to compare categorical variables, and t-tests (or non-parametric tests) were used to compare continuous variables between the early and advanced stages of LF. The Nutrient Analysis Template developed by the Food Science and Nutrition Department at the University of Ghana was used to estimate the nutrient composition of foods from the 24-hour recall. Random forest models were employed to identify key predictors of LF stage progression. Feature importance, reflecting the contribution of each variable to improving classification accuracy, was calculated, and the top predictors were visualized in a dot plot. Statistical significance was considered at p-value <  0.05 at 95% confidence level. The study employed CDC’s Epi Info® 7 for questionnaire design, data entry, and graphs generated with Microsoft Office 365 Excel. Analyses were conducted in R version 4.3.3, using packages such as random Forest and ggplot2 for machine learning and visualization.

## Results

### Socio-demographics of study participants

The study comprised 109 participants, 75.2% female and an average age of 50.72 ±  13.82. About half (50.4%) of the participants were engaged in agricultural activities and 18.3% were unemployed. Only 13.1% of the patients had wounds, and 12.8% had co-morbidities or experienced gastrointestinal symptoms. Table 2 shows the characteristics of the study participants.

**Table 2:** Characteristics of study participants.

Variables		n (%)	*p*-value
Age	Mean (SD)	50.72 (13.8)	
Gender	Male	27 (24.8)	<0.001
	Female	82 (75.2)	
Marital Status	Single	12 (11.0)	<0.001
	Married	66 (60.6)	
	Divorced/Separated	10 (9.2)	
	Widowed	21 (19.3)	
Education	Basic	103 (94.5)	<0.001
	Post-basic	6 (5.5)	
Occupation	Civil servant	1 (0.9)	<0.001
	Trader	13 (11.9)	
	Unemployed	20 (18.3)	
	Farmer	36 (33.0)	
	Fisher	19 (17.4)	
	Other (specify)	20 (18.3)	
Presence of wound	No	95 (87.2)	<0.001
	Yes	14 (12.8)	
Co-morbidities	No	95 (87.2)	<0.001
	Yes	14 (12.8)	
Physical Activities Categories	Low	6 (5.5)	<0.001
	Moderate	21 (19.3)	
	High	82 (75.2)	

### Anthropometry, body composition, and nutrition risk assessment

**Fig 1:**
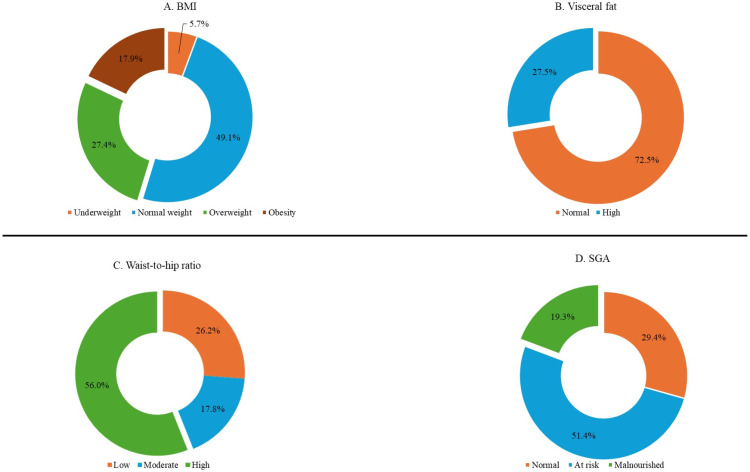
Anthropometric and body composition indices of study participants. This figure presents the distribution of anthropometric and body composition indices among study participants. Subfigure (A) shows the proportion of individuals classified as overweight or obese. Subfigure (B) depicts the percentage of participants with high visceral fat levels. Subfigure (C) illustrates the proportion of participants with high-risk waist-to-hip ratios. Subfigure (D) displays the distribution of participants at risk of malnutrition or already malnourished based on the Subjective Global Assessment (SGA) tool.

Anthropometric measurements, body composition, and SGA were used as part of the nutritional status assessment of participants. The results showed that nearly half, 45.3%, of the participants were either overweight or obese (A); about a quarter of the participants, 27.5%, had high visceral fat (B); more than half, 56.1%, were within the high-risk range for the waist-to-hip ratio (C); and majority, 70.7%, were either malnourished or at risk of being malnourished based on SGA (D), as shown in Fig 1.

### Nutrient deficiencies

#### Macronutrients and caloric intake.

**Fig 2:**
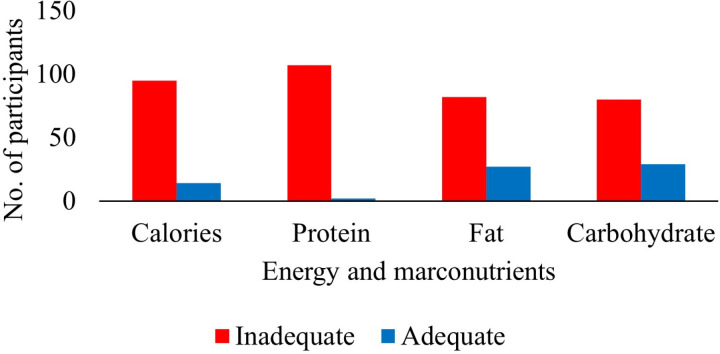
Calories and macronutrients consumption. This figure highlights the dietary macronutrient consumption and caloric intake among the study participants. It shows the percentage of individuals with inadequate intake of protein, fats, and carbohydrates, as well as the proportion of participants not meeting daily caloric requirements.

To identify specific nutrient deficiencies, their macronutrient and caloric intake were analyzed using AMDR for age and sex. It was observed that most of the patients had inadequate macronutrient intake, with 98.2% inadequate protein intake, 75.2% inadequate fat intake, and 73.4% inadequate carbohydrate intake. Caloric intake was also considered to determine the population’s health status. However, it was observed that most of the participants, 87.2%, had inadequate caloric intake, as shown in Fig 2.

#### Micronutrients.

**Fig 3:**
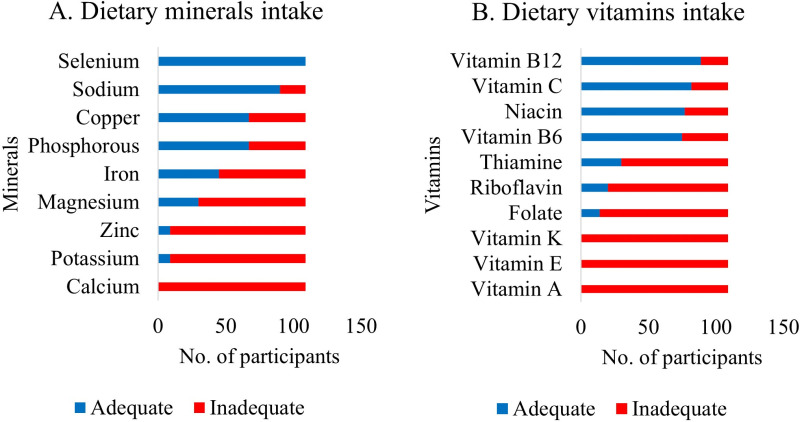
Micronutrient distribution among study participants. The figure illustrates the prevalence of micronutrient inadequacies among study participants. Subfigure (A) represents deficiencies in essential minerals such as calcium, potassium, and zinc. Subfigure (B) presents the prevalence of vitamin deficiencies, including vitamins A, C, E, K, and B-complex vitamins.

In the assessment of dietary micronutrient intakes, inadequacies of certain minerals were noted, with Calcium, Potassium, and Zinc being the highest at 100%, 91.7%, and 91.7%, respectively. Iron at 58.7% and Magnesium at 72.5% were also inadequate in their diets. However, Selenium at 100%, Sodium at 82.6%, Copper at 61.5%, and Phosphorus at 61.5% were seen to be adequate, as shown in Fig 3A.

There was a wide range of inadequacies among vitamin intakes, with Vitamin A, Vitamin E, Vitamin K, Thiamine, Folate, and Riboflavin having the highest recorded inadequacies among the participants at 100%. Moreover, Vitamin B12, Vitamin C, Niacin, and Vitamin B6 recorded the highest adequacies, with 81.7%, 75.2%, 70.6%, and 68.8%, respectively, as shown in Fig 3B.

### High prevalence of anemia among study participants

**Fig 4:**
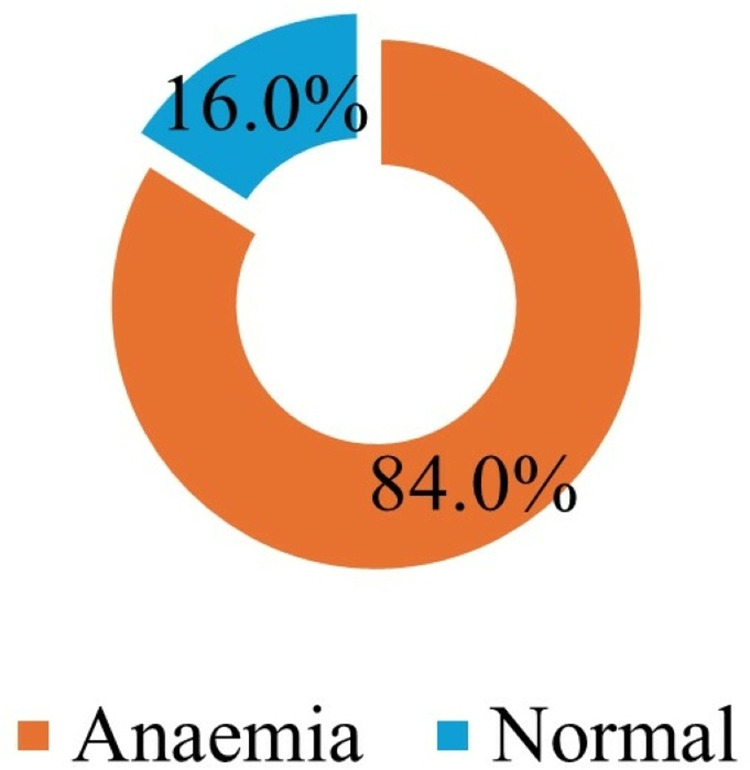
Prevalence of anemia among the study participants. This figure depicts the distribution of anemia among the study participants. It shows the percentage of individuals with hemoglobin levels below the normal threshold, indicating a high prevalence of anemia in the study population.

In the estimation of the hemoglobin levels of the participants, it was observed that out of the 109 participants, 89 (84.0%) were anemic, as shown in Fig 4.

### Factors associated with nutrition status (SGA)

In assessment of the factors associated with the nutritional status of participants (SGA), no statistically significant association was identified, as shown in Table 3 below.

**Table 3:** Factors associated with nutritional status of participants.

Variables		SGA				
		Normal	At risk	Malnourished	X2 (df)	p-value
Sex	Male	10	11	6	1.674 (2)	0.433
	Female	22	45	15		
Marital status	Unmarried	10	23	10	1.549 (2)	0.461
	Married	22	33	11		
Education	Up to basic	29	54	20		0.554
	Post-basic	3	2	1		
Comorbidity	No	27	51	17		0.412
	Yes	5	5	4		
Alcoholic	No	19	36	15	0.802 (2)	0.670
	Yes	13	20	6		
Smoker	No	31	56	21		0.486
	Yes	1	0	0		
Anemia	Normal	6	7	4		0.641
	Anemic	26	49	17		

### Decision tree

**Fig 5:**
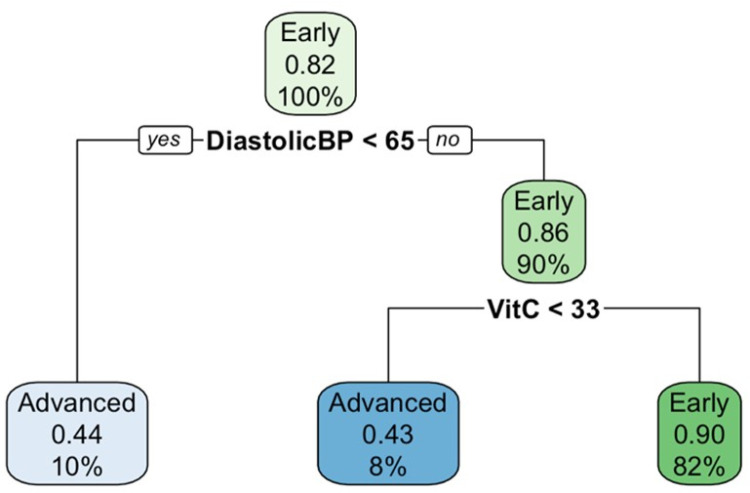
Decision tree showing the influence of diastolic blood pressure and vitamin C levels on the progression of LF. The decision tree model identifies key predictors influencing the progression of lymphatic filariasis (LF). It highlights the role of diastolic blood pressure and vitamin C levels in determining disease severity, with thresholds indicating an increased likelihood of progression from early to advanced stages.

The decision tree model identified diastolic blood pressure (DiastolicBP) and vitamin C (VitC) levels as key predictors of LF progression. Participants with diastolic blood pressure less than 65 mmHg had an 82% probability of remaining in the early stages of LF. Otherwise, vitamin C less than 33 mg/dL was associated with a higher risk of progression to advanced stages, with a probability of 43%. On the other hand, participants with higher vitamin C levels, at least 33 mg/dL, had a 90% probability of remaining in the early stages, as shown in Fig 5.

### Random forest variable importance plot

**Fig 6:**
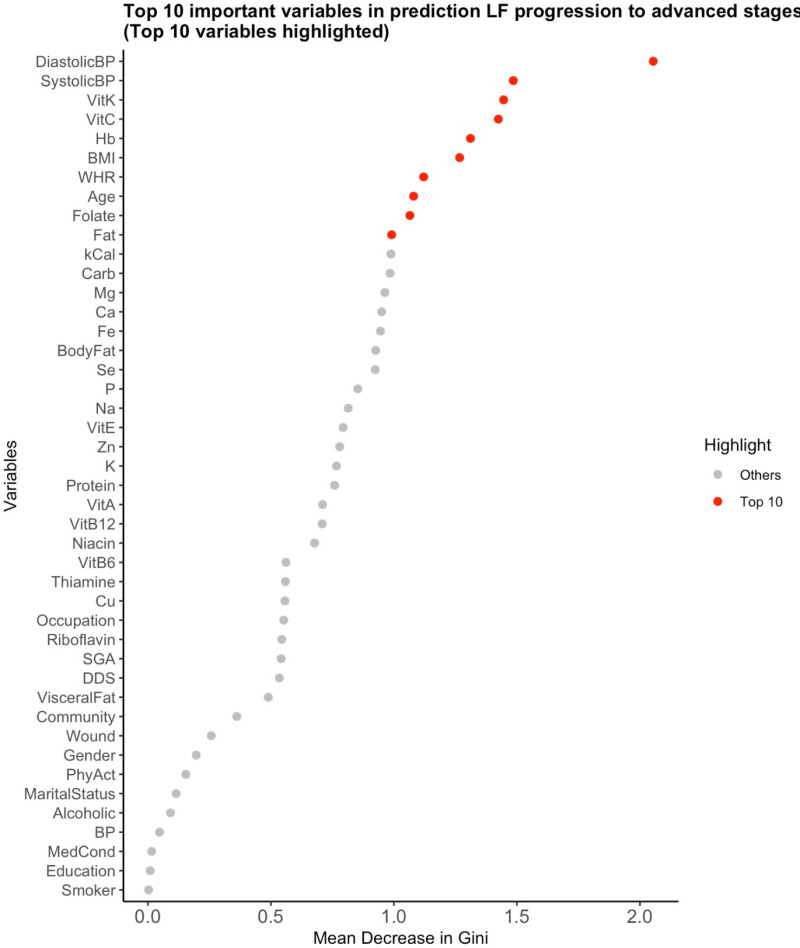
Random forest plot showing the importance of predictors (top 10) for classifying LF progression based on the Mean Decrease in Gini index. This figure presents the results of the random forest model, ranking the most important variables in predicting LF progression. Elevated blood pressure, vitamin C, and vitamin K emerge as the strongest predictors, along with other factors such as BMI, anemia status, folate levels, and age.

The random forest plot identified key predictors for LF progression (early vs. advanced) based on the Mean Decrease in the Gini index. Elevated blood pressure, Vitamins C and K, are the most important variables, as shown in Fig 6. Other predictors include BMI, anemia (Hb), folate, and age. Thiamine, copper (Cu), and visceral fat contribute less to the classification process and disease progression. The decision tree and random forest emphasize the influence of vitamins C and K and blood pressure in distinguishing early from advanced LF stages.

## Discussion

Significant strides have been made globally to eradicate LF as a public health concern. However, people living with LF pathologies have their quality of life reduced by the chronicity of the condition. Understanding the impact of nutritional deficiencies on immune function, disease severity, and progression of LF, although limited, is critical in ensuring the optimum management of LF-associated pathology and complications. With the impact of nutritional deficiencies on immune function known, nutritional deficiencies in LF patients are stipulated to further complicate the treatment and management of LF morbidities. This study identified vitamins C and K, blood pressure (systolic and diastolic), and hemoglobin as the most important factors of disease progression in LF.

Vitamin C is pivotal in immune function, primarily as an antioxidant and aiding in various innate and adaptive immune response processes. Studies have demonstrated that sufficient Vitamin C levels help boost the immunological response to infections such as LF. Deficiency in Vitamin C has been associated with decreased immunological responses, which may worsen the severity of LF and contribute to disease progression [19]. Furthermore, individuals with Vitamin C deficiency are more susceptible to infections – contributing to secondary bacterial and fungal infections in LF wounds [20]. In the context of LF, filarial infections induce inflammation within the lymphatic system, which not only causes oxidative stress but also leads to damage of lymphatic vessels and subsequent lymphatic dysfunction, thereby predisposing patients to recurring bacterial infections [21]. Therefore, low levels of Vitamin C may lead to the accumulation of reactive oxygen species, resulting in increased oxidative stress and contributing to the severity of the disease [22]. Vitamin C may mitigate oxidative stress in LF patients, potentially slowing disease progression. Given the chronic inflammation in LF, Vitamin C’s antioxidant properties are crucial in reducing oxidative damage and supporting lymphatic repair. Its deficiency may impair tissue healing and exacerbate disease progression, especially in patients with LF wounds.

Vitamin K has been demonstrated to contribute to cardiovascular health by influencing vascular function, potentially impacting lymphatic circulation and drainage [23]. The interplay between these vitamins suggests that maintaining adequate levels of both is crucial for individuals at risk of or suffering from LF, as they help reduce oxidative damage and support vascular integrity. The role of Vitamin K in vascular function is especially relevant in LF, as maintaining proper lymphatic and vascular circulation is key to managing disease progression. Deficiency in Vitamin K could accelerate lymphatic damage, contributing to worsening symptoms.

Blood pressure is another critical factor associated with LF progression. Elevated blood pressure can lead to vascular alterations that aggravate lymphatic dysfunction, a hallmark of LF pathology [24]. Increased systolic and diastolic blood pressure likely contribute to LF progression by worsening lymphatic congestion and impairing fluid clearance. Hypertension management could be crucial in slowing down disease progression in LF patients. Chronic hypertension induces systemic inflammation, which can impair immunological responses and facilitate the progression of infections, including LF [25]. This relationship underscores the importance of monitoring cardiovascular health in LF patients, as managing blood pressure may help slow disease progression.

Anemia, often indicated by low hemoglobin levels – iron depletion and deficiency – can impair immune function and increase susceptibility to infections [26]. In LF-endemic regions, individuals with anemia may experience more severe clinical outcomes of the disease due to their compromised immune status. Studies have indicated that individuals with higher hemoglobin levels tend to have better immune responses and lower rates of severe LF complications [27–29]. This could be attributed to improved oxygen and nutrient delivery to tissues, which allows better lymphatic function and reduces the risk of secondary infections that can exacerbate LF pathologies [30]. Thus, addressing anemia through nutritional interventions and medical treatment could be vital to LF management strategies. Folate deficiency could lead to impaired immune responses, increasing susceptibility to more advanced stages of the disease.

Additionally, folate deficiency (87.2%) could contribute to anemia since it is required for nucleic acid synthesis necessary for red blood cell production. Folate deficiency may also impair immune cell production and function, potentially contributing to the advancement of LF. The role of folate in both immune regulation and the prevention of anemia makes it a key factor in disease progression.

The role of micronutrients in infectious diseases is well-documented, with several studies indicating that deficiencies can lead to impaired immune responses and increased susceptibility to infections [12,22,31]. For instance, in this study, micronutrients such as Vitamin A, zinc, and iron, with 100%, 91.7%, and 72.5% deficiencies, respectively, have been shown to modulate immune function and influence the course of various infectious diseases [32]. These findings corroborate previous research demonstrating the potential of nutritional interventions in managing LF-associated morbidity. Douglass et al. [33] showed that an enhanced care package, including dietary modifications, improved outcomes in individuals with lymphedema. This supports the observed importance of micronutrient intake, particularly vitamins C and K, in LF progression. Integrating dietary improvements into LF care programs could offer a cost-effective approach to mitigating disease severity and enhancing patient outcomes. In LF, micronutrient deficiencies may exacerbate the disease by impairing the host’s ability to mount an effective immune response against the filarial parasites [34]. Although high malnutrition risk (70.7%) was identified in this study, it was not implicated as a major contributor to disease progression. Albeit being a subjective assessment, it provides insight for further studies and interventional strategies.

The importance of Vitamin C and K, along with blood pressure, suggests that oxidative stress and cardiovascular health are critical factors in the progression of lymphatic filariasis [35–37]. These findings are consistent with existing literature highlighting micronutrients’ role in infectious diseases and their impact on immune function. Furthermore, the interplay between nutrition, immune function, and disease progression underscores the importance of a holistic approach to managing LF, which includes addressing nutritional deficiencies alongside traditional medical treatments [38].

Other significant predictors observed include obesity (17.9% in this study) and age. Obesity predisposes individuals to chronic inflammation – worsening immune function and fostering lymphatic dysfunction [39,40]. Excess body fat, particularly visceral fat, can intensify inflammation and impair lymphatic drainage in LF patients, potentially accelerating disease progression. This underscores the importance of addressing obesity as part of LF management strategies. Age, a well-established predictor of several morbidities, is often linked to increased severity of clinical manifestations. This relationship may be attributed to the cumulative effects of chronic infections and the natural decline in immune function that occurs with aging [41].

Further analysis in evaluating the nutritional risk in relation to sociodemographic factors, medical history (comorbidities), and lifestyle habits, such as alcohol consumption and smoking, revealed no statistically significant associations with nutritional risk. This suggests that other factors, such as dietary intake, physical activity levels, and underlying metabolic conditions, may play a more significant role in determining nutritional status in this population. The management of lymphatic filariasis (LF) is multifaceted. This study suggests that nutritional interventions focusing on micronutrient supplementation, particularly Vitamin C and K, and blood pressure management could be critical in slowing disease progression. Foods rich in antioxidants and micronutrient supplementation may enhance immune function and reduce oxidative stress, which are pivotal in controlling the severity of LF. Nutritional intervention could be developed to supplement current management modules considering local contexts.

The use of a cross-sectional design limits the ability to infer causality. The recall-based dietary assessment employed in this study is less objective than the invasive biochemical approach. Of note, year-round dietary variations could be considered since farming in Ghana is dependent on rainfall, affecting the availability of produce in different seasons of the year. The powerful random forest models may be prone to overfitting, considering the small size of this study’s dataset. Further matched case-control studies can be explored to identify the differences between LF patients and the general population in endemic areas or multicenter longitudinal studies to investigate the causal relationships between nutritional status, particularly micronutrient levels, and LF progression. Future studies should apply machine learning techniques to larger, multicentric datasets. This expansion will enhance the generalizability of findings and allow for identifying predictors across diverse populations and settings.

## Conclusions

This study identified micronutrient deficiencies, especially vitamins C and K, and blood pressure as the key predictors of LF progression. Other factors included anemia, folate deficiency, and obesity. These findings suggest that interventions focusing on improving vitamin C and K intake or supplementation and managing blood pressure are likely to slow LF progression and improve the quality of life of people living with LF.

## Supporting information

S1 File Nutritional and Socio-Demographic Assessment of Lymphatic Filariasis Patients in Ghana.
This dataset contains socio-demographic, clinical, and nutritional data from individuals affected by lymphatic filariasis (LF) in Ghana. It includes information on age, gender, community, marital status, education, occupation, disease stage, wound presence, and comorbid conditions. It also provides dietary diversity scores (DDS), vitamin levels (B12, A, E, K), and their categorical classifications.
(CSV)

S2 Checklist Checklist.
(DOCX)
